# Differences in the thermal physiology of adult Yarrow's spiny lizards (*Sceloporus jarrovii*) in relation to sex and body size

**DOI:** 10.1002/ece3.1297

**Published:** 2014-10-20

**Authors:** Martin S Beal, Matthew S Lattanzio, Donald B Miles

**Affiliations:** Department of Biological Sciences, 107 Irvine Hall, Ohio UniversityAthens, Ohio, 45701

**Keywords:** Critical thermal temperature, physiological state, preferred body temperature, sexual dimorphism, thermal performance curve

## Abstract

Sexual size dimorphism (SSD) is often assumed to reflect the phenotypic consequences of differential selection operating on each sex. Species that exhibit SSD may also show intersexual differences in other traits, including field-active body temperatures, preferred temperatures, and locomotor performance. For these traits, differences may be correlated with differences in body size or reflect sex-specific trait optima. Male and female Yarrow's spiny lizards, *Sceloporus jarrovii*, in a population in southeastern Arizona exhibit a difference in body temperature that is unrelated to variation in body size. The observed sexual variation in body temperature may reflect divergence in thermal physiology between the sexes. To test this hypothesis, we measured the preferred body temperatures of male and female lizards when recently fed and fasted. We also estimated the thermal sensitivity of stamina at seven body temperatures. Variation in these traits provided an opportunity to determine whether body size or sex-specific variation unrelated to size shaped their thermal physiology. Female lizards, but not males, preferred a lower body temperature when fasted, and this pattern was unrelated to body size. Larger individuals exhibited greater stamina, but we detected no significant effect of sex on the shape or height of the thermal performance curves. The thermal preference of males and females in a thermal gradient exceeded the optimal temperature for performance in both sexes. Our findings suggest that differences in thermal physiology are both sex- and size-based and that peak performance at low body temperatures may be adaptive given the reproductive cycles of this viviparous species. We consider the implications of our findings for the persistence of *S. jarrovii* and other montane ectotherms in the face of climate warming.

## Introduction

Sexual size dimorphism (SSD) likely reflects adaptive effects of differing selective agents acting on each sex (Selander 1966; Zamudio 1998; Blanckenhorn 2005). Elucidating the physiological and ecological consequences of SSD provides insight into the proximate and ultimate mechanisms underlying the evolution and maintenance of phenotypic variation (Badyaev 2002; Cox and John-Alder 2007). Males and females of species that exhibit SSD are often dimorphic in other phenotypic traits, including behavior, energetics, physiological performance, and ecology (Cullum 1998; Irschick and Garland 2001). For example, Lailvaux et al. (2003) demonstrated that male and female flat lizards (*Platysaurus intermedius wilhelmi*) differ in sprint capacity, a trait used as a proxy for fitness. Similarly, although male striped plateau lizards (*Sceloporus virgatus*) exhibit higher metabolic rates, female lizards have longer activity periods, consume more food, and exhibit greater assimilation efficiency (Merker and Nagy 1984). In giant petrels (*Macronectes* spp.), females exhibit longer search distances (a measure of foraging effort) and flight speed than males, yet males gain greater proportional mass than females while foraging due to their larger size (González-Solís et al. 2000).

Many physiological traits are affected by both body size and temperature (Huey 1982; Schmidt-Nielsen 1984). Therefore, in species that exhibit SSD, differences in physiological traits between males and females may be a consequence of body size (Lailvaux 2007). Previous research shows that body size accounts for differences between the sexes in numerous traits, including temperature selection (Zari 1998), foraging behavior (Houston and Shine 1993), and performance (Cullum 1998; Lailvaux and Irschick 2007). Alternatively, males and females may differ in traits that are uncorrelated with body size (Lailvaux 2007). For example, Butler et al. (2000) showed that exploitation of different microhabitats by male and female *Anolis* lizards could not be attributable to size differences between the sexes. Understanding the physiological and ecological consequences of SSD requires a determination of the effects of both sex and body size on variation in these traits (Blanckenhorn 2005).

Lizards are a model organism for evaluating the consequences of SSD on thermal physiology. There is a substantial amount of data on thermal physiology, including body temperature, preferred temperature, and the effects of temperature on physiological performance. Although the focus of many of these studies typically involves species that exhibit SSD (Angilletta et al. 2002a; Lailvaux et al. 2003; Lailvaux and Irschick 2007; Schuler et al. 2011), scant attention has been paid to the effects of body size and sex on thermal physiology (but see Lailvaux 2007). For some lizard species, body temperature differences between males and females may be a consequence of either body size or sex-specific differences in trait optima (Stevenson 1985; Woolrich-Piña et al. 2012). Males and females of species exhibiting SSD may therefore differ in thermal physiology that is unrelated to size. Because body temperature influences multiple traits including activity patterns (Grant and Dunham 1988), behavior (Bennett 1980), and metabolism (Chen et al. 2003), sexual differences in body temperature may result in sex-specific differences in a myriad of performance traits, including sprint speed, stamina, and assimilation efficiency. There is likely not a single body temperature at which all physiological processes are optimized (Huey 1982). Therefore, ectotherms should use behavioral and physiological means to regulate their body temperature to satisfy current energetic or maintenance demands (Hertz et al. 1988, 1993; Brown and Griffin 2005). For example, individuals in a fasted state generally prefer a lower body temperature in a laboratory gradient than when recently fed (Brown and Griffin 2005), presumably to avoid higher metabolic costs of elevated body temperatures.

In addition, locomotor performance is critical for lizards because it has direct ties to survival (Garland and Losos 1994; Miles 1994, 2004). Measuring locomotor performance of a lizard at different temperatures provides an estimate of optimal temperature for performance as well as the shape or form of the sensitivity of performance to variation in body temperature (e.g., Angilletta 2006; Zajitschek et al. 2012). If males and females exhibit SSD and diverge in other traits, including body temperature, then both the magnitude of variation in preferred body temperatures with respect to similar energetic states and the relationship between body temperature and performance capacity may also differ between the sexes.

Here, we test whether male and female Yarrow's spiny lizards (*Sceloporus jarrovii*) exhibit differences in field-active body temperature, preferred (selected) temperatures, and thermal sensitivity in locomotor performance. Yarrow's spiny lizards exhibit male-biased SSD. Males and females also show annual variation in territorial behavior that should influence the range of body temperatures each sex experiences, potentially contributing to sex-specific differences in thermal physiology (Moore and Marler 1987). Preliminary data from our study site revealed that male and female lizards differ in body temperature (*T*_b_) unrelated to variation in body size. In this study, we determined the preferred body temperature (*T*_pref_) of males and females in a thermal gradient. We measured the *T*_pref_ for each lizard when fed and fasted. An earlier study of male *S. jarrovii* showed no difference in *T*_pref_ when fed or fasted (Schuler et al. 2011), and we therefore predict that only females will have lower values for *T*_pref_ when fasted. Next, we compared male and female thermal sensitivity in locomotor performance. Body size has been shown to affect performance capacity and interact with sensitivity of performance to different *T*_b_s (Garland 1994). The form of the thermal performance curve for stamina should also differ between male and female *S. jarrovii*. Males only defend territories during June–August. However, both sexes defend territories during the fall breeding season (September–November). We predicted both sexes would exhibit a similar performance curve (driven by fall activity), but males should exhibit a higher peak in performance than females (driven by year-round territoriality). Finally, because body size may also affect performance capacity, we also predict that larger lizards will have greater stamina at the optimal performance temperature (*T*_opt_).

## Materials and Methods

### Field data

Our first goal involved determining whether males and females differed in body size and *T*_b_. Previous studies have established male-biased SSD in *S. jarrovii*. However, sex-specific differences in *T*_b_ have not been observed before in *S. jarrovii*. We used the field data to establish whether *T*_b_ differences are a consequence of body size or reflect broader differences in thermal physiology between males and females, such as differences in microhabitat selection. We captured adult male (*n* = 25) and female (*n* = 34) *S. jarrovii* from Miller Canyon in the Huachuca Mountains, Cochise County, Arizona, in June 2012. Large boulders and Ponderosa Pine are the dominant structural features of the study site. We captured lizards using nooses and recorded the *T*_b_ (°C) of each lizard within 10 sec using a Raytek infrared thermometer (Fluke Corporation, Everett, WA) pressed firmly against the cloaca. After obtaining the *T*_b_ of the lizards, we measured snout–vent length (SVL, in mm) of all lizards and captured microhabitat.

### Estimation of thermal traits

Our test of the differences in thermal physiology in males and females used a subsample of *S. jarrovii* (males, *n* = 10; females, *n* = 14) from the field captures. We transported these lizards to a laboratory facility at the Appleton-Whittell Research Ranch near Elgin, AZ. Each lizard was housed in a separate 5.7 L terraria (27.9 cm L × 17.8 cm W × 12.7 cm H, Frey Scientific, Nashua, NH). The room had lights suspended above each terrarium connected to a timer to mimic the local photoperiod (15:9 h light:dark cycle). We measured SVL to the nearest 0.1 mm using callipers and mass to the nearest 0.1 g using a Pesola™ scale. All lizards were provided water ad libitum and fed two adult (∼2.54 cm total length) domestic crickets (*Acheta domestica*) every 3 days after capture. Uneaten crickets were removed from each terrarium after 12 h.

### Thermal preference

We estimated male and female *T*_pref_ in a thermal gradient (Hertz et al. 1993; Schuler et al. 2011). The thermal gradient measured 1.22 m × 0.15 m × 0.25 m (L × W× H). A heat source (heat pad) placed at one end of the gradient maintained a temperature of 48°C. We chilled the opposite end of the gradient using ice packs to maintain a temperature of 22°C. Daylight simulation lamps suspended above the gradient provided uniform lighting. At the start of each trial, we recorded the initial *T*_b_ of each lizard and then placed the lizard in the centre of the gradient facing perpendicular to either end. We measured *T*_b_ after 30 min, to allow lizards an opportunity to acclimate to the gradient. We subsequently obtained additional measurements every 15 min thereafter. Our estimate of *T*_pref_ comprised 90 min in the thermal gradient and a total of five *T*_b_ values. We used the same infrared thermometer (pressed against the lizards' cloaca) as we used to collect field data. The mean *T*_b_ over the 90-min period was our estimate of a lizards' *T*_pref_ (Schuler et al. 2011).

We estimated the dependence of *T*_pref_ on nutritional state using consecutive trials (Schuler et al. 2011). The *T*_pref_ of all lizards was measured first in a fasted state and then in a fed state. Fasted states involved starving lizards for a 48-h period prior to measuring *T*_pref_ in the thermal gradient (Segall et al. 2013). All lizards defecated during this period. We returned lizards to their terraria at the end of the fasted trial and allowed them to rest undisturbed for at least 1 week before measuring their *T*_pref_ in a fed state. The *T*_pref_ of each lizard in a recently fed state was estimated approximately 30 min after lizards were provided with food (two *A. domestica*). We confirmed that each lizard consumed both crickets prior to initiating the thermal preference trial.

### Thermal tolerance: CT_min_ and CT_max_

Critical thermal limits delimit the range of temperatures beyond which an organism cannot function (i.e., thermal tolerance zone) and as such are an important component of the thermal physiology of a species (Cowles and Bogert 1944; Angilletta et al. 2002b). We estimated the critical minimum (CT_min_) and maximum (CT_max_) of male and female *S. jarrovii* over a 2-day period following completion of our experiment using a different sample (*n* = 5 of each sex) of lizards from the study population. We collected these individuals in June 2012. To estimate CT_min_, we placed a lizard in an insulated container which resulted in a cooling rate of approximately 1–2°C per minute. We used the *T*_b_ at which a lizard lost its righting response as our estimate of CT_min_. Our estimate of CT_max_ involved placing lizards in a container with a lamp suspended above to yield a heating rate of 1–2°C per minute. The *T*_b_ of a lizard upon loss of righting response was used as the CT_max_. We checked for loss of righting response every minute in both experiments.

### Thermal sensitivity of performance

Stamina is a measure of whole-organism performance that is assumed to affect the ability of a lizard to defend territories, exhibit display behavior, and escape a predator (e.g., Garland and Losos 1994; Sinervo et al. 2000). Our method of measuring stamina involved chasing a lizard around a circular racetrack (Clobert et al. 2000; Robson and Miles 2000). The racetrack has an external and internal diameter of 100 and 60 cm, respectively, and a track width of 20 cm. A 1-cm layer of sand served as a substrate. We encouraged each lizard to run by lightly tapping on its tail (Clobert et al. 2000), and we recorded the time until a lizard became fatigued (seconds) as our estimate of stamina. We used the loss of a righting response as our measure of fatigue as in past studies (Robson and Miles 2000). Previous work indicates this method of quantifying is repeatable (Garland et al. 1990; Robson and Miles 2000).

We estimated the thermal performance curve for stamina of each lizard across seven temperatures in a random sequence (32°C, 26°C, 20°C, 29°C, 38°C, 23°C, and 35°C). We raced lizards at one temperature per day, allowing them 48 h to rest in between races (Angilletta et al. 2002a). Approximately 1 h prior to a trial, each lizard was acclimated to the trial temperature in a separate enclosure. Heating pads, overhead lights, and/or ice packs were used to achieve the desired ambient and substrate temperatures in these enclosures. The racetrack was also equilibrated to each race temperature prior to initiating each trial. A trial was initiated when the lizards' *T*_b_ was within ±0.4°C of the target temperature. We recorded the *T*_b_ at which stamina was maximum for each lizard as our estimate of *T*_opt_.

### Data analyses

All statistical analyses were conducted within the r software environment (R Development Core Team 2012). We used a t-test to compare body size and body condition (residuals of a regression of mass on SVL) between male and female *S. jarrovii*. We compared the field *T*_b_ of male and female lizards using an ANCOVA. The *T*_pref_ of males and females in a laboratory gradient when fed or fasted was compared by sex (male or female) using a repeated-measures ANCOVA. Body size (SVL) was included as the covariate in both ANCOVAs.

We estimated the shape of the thermal performance curves of male and female lizards using generalized additive mixed models (GAMM) implemented using the function “gamm” in the mgcv package (Wood 2006). We included SVL as a covariate in our GAMM models to determine its effect on the shape and location of each curve. GAMMs are ideal for detecting differences in thermal performance curves between groups (e.g., sex) that may be attributed to differences in maximal performance or the shape of the curve across all measured temperatures, or both. We initially tested four models: one with a single curve for both sexes (same curve shape and *y*-axis values), one with a distinctly shaped curve for each sex (different curve shape but same *y*-axis values), one with a distinctly shaped curve and different *y*-axis values, and one where males and females share the same performance curve but different maximal performance (curve *y*-axis values), following Zajitschek et al. (2012). We employed a cubic spline smoothing method and fixed knots to the CT_min_, the seven temperatures at which stamina was measured, and CT_max_ (nine total knots). A Gaussian distribution of residuals was assumed in these models, and we used the Akaike information criterion (AIC) to evaluate model fit (Burnham and Anderson 2002). We present results from the best-fit model in Results.

We used *t*-tests to compare *T*_opt_ (experimental temperature at which stamina was maximized for each individual) to *T*_pref_ in the laboratory gradient (fasted only) for male and female *S. jarrovii*. We estimated maximal performance breadth as the range of temperatures at which lizards could perform at 95% of their maximum performance capacity (Du et al. 2000; Angilletta et al. 2002a). We compared the 95% performance breadth between males and females using a t-test. All means are presented as ±1.0 standard error (SE).

## Results

### Field data on SSD, body condition, and field-active body temperatures

**Figure 1:**
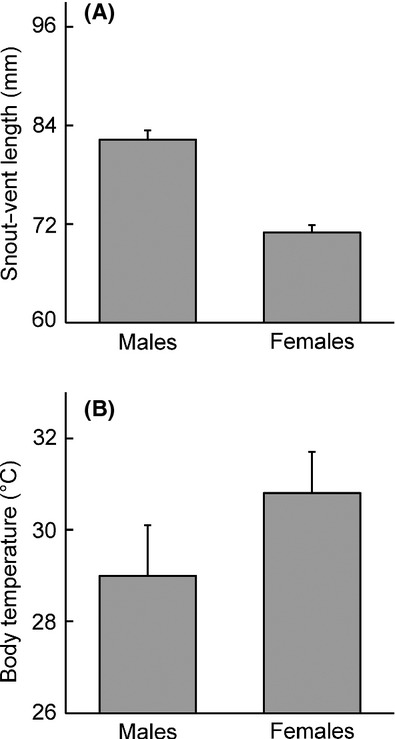
(A) Mean body size (snout–vent length, mm) for male and female *Sceloporus jarrovii*. Males were 16% larger than females. (B) Mean body temperature (*T*_b_, °C) for males and females. Sample sizes: males, *n* = 25; females, *n* = 34.

Male *S. jarrovii* were larger than females in both SVL (males, 86.3 ± 1.6 mm; females, 75.2 ± 1.9 mm) and mass (males, 23.5 ± 1.5 g; females, 15.4 ± 1.4 g). This population of *S. jarrovii* exhibits male-biased SSD in body size with males being 16% larger than females (*t* = −8.12, df = 51.925, *P* < 0.001; Fig. 1A). Males and females showed no difference in body condition (*t* = −0.04, df = 19.415, *P* = 0.973).

In addition, female *S. jarrovii* exhibited a higher mean *T*_b_ than males in this population (males: 29 ± 0.5°C, females 30.8 ± 0.3°C; ANCOVA, *F*_1,56_ = 8.92, *P* = 0.004; Fig. 1B). There was no effect of SVL on *T*_b_ (*F*_1,56_ = 0.05, *P* = 0.829).

### Thermal preference in a laboratory gradient and thermal tolerances

**Figure 2:**
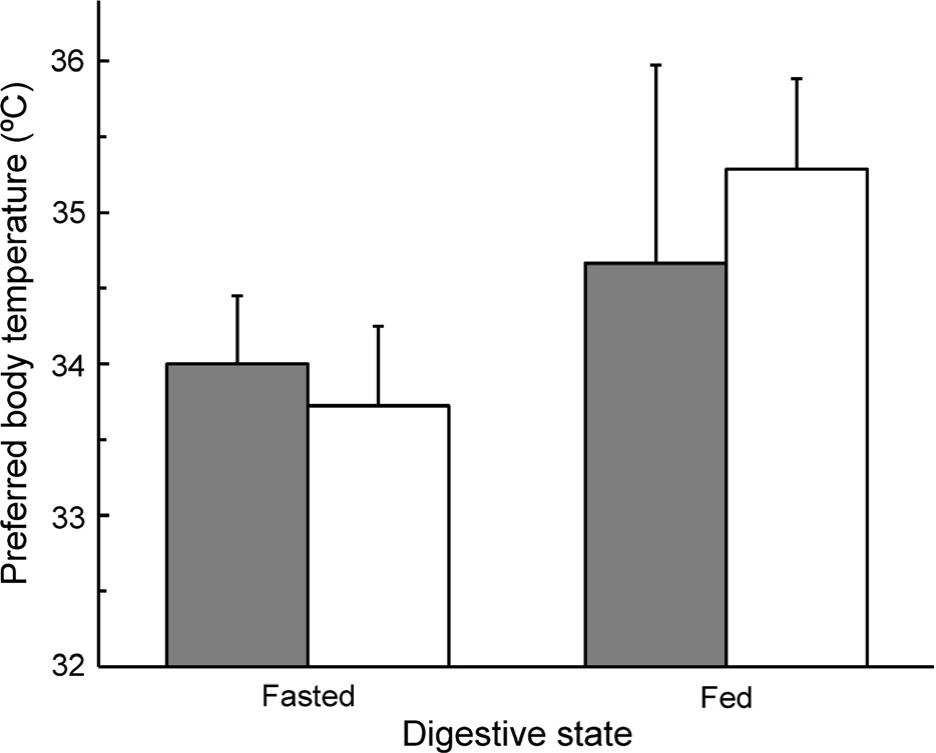
Preferred body temperatures (*T*_pref_, °C) of adult male (*n* = 10, gray bars) and female (*n* = 14, white bars) *Sceloporus jarrovii* measured when fasted and fed. Fasted lizards had food withheld for 48 h prior to measurement of *T*_pref_. Fed lizards were tested within 30 min of an observed feeding event. Bars are +1.0 standard error (SE).

Male and female *S. jarrovii* exhibited differences in *T*_pref_ between fed and fasted states (Table 1). Specifically, fed and fasted male lizards exhibited similar values for *T*_pref_ (fed: 34.7 ± 1.3°C; fasted: 34 ± 0.5°C; *F*_1,8_ = 0.24, *P* = 0.64; Fig. 2). Body size was unrelated to *T*_pref_ (*F*_1,8_ = 1.95, *P* = 0.2). In contrast, female lizards had higher values for *T*_pref_ when fed (fed: 35.3 ± 0.6°C; fasted: 33.7 ± 0.5°C; *F*_1,12_ = 10.03, *P* = 0.008). Body size had no effect on female *T*_pref_ (*F*_1,12_ = 0.18, *P* = 0.678). The critical thermal limits of male and female *S. jarrovii* overlapped (CT_min_, males: 13.4 ± 0.2°C; females: 13.8 ± 0.5°C; CT_max_, males: 39.1 ± 0.8°C; females: 39.3 ± 1.7°C) (Table 1).

**Table 1:** Thermal physiology of male (*n* = 10) and female (*n* = 14) *Sceloporus jarrovii*, including preferred body temperature when fasted (*T*_pref_ below) and fed (*T*_pref_ fed below), thermal tolerances (CT_min_ and CT_max_, respectively), and optimal performance temperature (*T*_opt_) for stamina. Values are mean (95% confidence interval). See Materials and Methods for variable definitions

Variable	Males	Females
*T*_pref_ (°C)	34 (33.1–34.9)	33.4 (32.4–34.5)
*T*_pref_fed (°C)	34.7 (32.1–37.2)	35.3 (34.1–36.5)
CT_min_ (°C)	13.4 (13.2–13.6)	13.8 (13.3–14.3)
CT_max_ (°C)	39.1 (38.3–39.9)	39.3 (37.6–41)
*T*_opt_ (°C)	28.4 (26.1–30.7)	26.4 (24.5–28.4)

Note: *N* = 5 for CT_min_ and CT_max_ for each sex.

### Thermal sensitivity of performance

**Figure 3:**
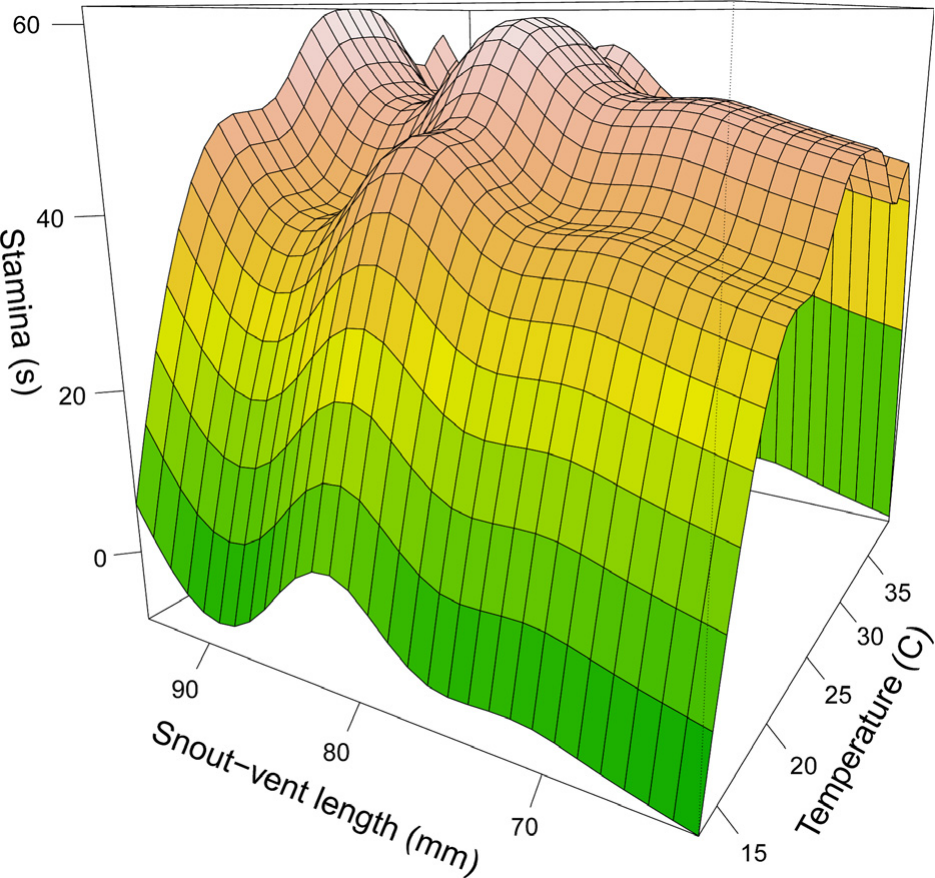
Effect of temperature (°C) and body size (snout–vent length, mm) on the thermal performance curve for stamina (sec) of adult *Sceloporus jarrovii* (*N* = 24). Note: shading on curve is for illustrative purposes only.

Male *S. jarrovii* exhibited a narrower (but overlapping) 95% performance breadth (mean [CI]: 28.1 [1.5]—31.7 [2.4]°C) than females (25.8 [1.1]—31.8 [1.4]°C). In addition, females exhibited a lower *T*_opt_ than male lizards (26.4 vs. 28.4°C, see Table 1). However, we detected no effect of sex on the thermal performance curve of stamina (Table 2). Removal of SVL as a covariate resulted in a significantly poorer model fit (Table 2). Our results support a significant effect of SVL on thermal performance curve shape (Table 2; Fig. 3). Finally, the *T*_pref_ selected by both males and females when fasted was higher than their *T*_opt_ for stamina (males: *t* = 3.68, df = 9, *P* = 0.005; females: *t* = 5.78, df = 13, *P* < 0.001) (see also Table 1).

**Table 2:** Results of a generalized additive mixed model describing the thermal sensitivity of stamina for adult *Sceloporus jarrovii*. Model selection involved an information-theoretic approach

Model	Syntax	AIC	ΔAIC	*w*_*i*_(AIC)
M0	∼s(Temperature)	1661.4	10.4	0.004
M1A	∼s(Temperature) + s(SVL)	1651	0	0.726
M1B	∼s(Temperature) + s(SVL) + factor(Sex)	1653	2	0.269
M2A	∼s(Temperature, by = Sex) + s(SVL)	1681.1	30.1	0
M2B	∼s(Temperature, by = Sex) + s(SVL) + factor(Sex)	1683.1	32.1	0

Models are described in Materials and Methods. Here, ΔAIC = [AIC_*i*_ − min(AIC)] and *w*_*i*_(AIC) = rounded Akaike weights calculated using the function “Weights” in the MuMIn package in R (Barton 2013).

## Discussion

The expression of SSD in a species is a consequence of developmental processes and selection favoring different fitness optima between males and females. An underexplored aspect of SSD is whether size differences between the sexes may be associated with divergence in other physiological traits. Adult *S. jarrovii* exhibit male-biased SSD in body size (Cox 2006; Cox and John-Alder 2007), and male and female lizards also differ in behavior throughout the year (i.e., territoriality, Moore and Marler 1987). In the Huachuca Mountains population of *S. jarrovii*, males are 16% larger than females, which is similar to the maximum estimates for SSD in this species (18%, see Cox 2006; see also Table S1). Females had a higher *T*_b_ than males even though both sexes utilize similar microhabitats (boulders or trees) throughout the day in this population (MSL, unpublished data). Only female *S. jarrovii* selected lower values of *T*_pref_ when fasted. However, both sexes shared a common thermal performance curve with *T*_opt_ for stamina occurring at *T*_b_s lower than their *T*_pref_. As in other studies, larger lizards had greater stamina (Miles et al. 2001). Our results suggest that male and female differences in thermal physiology are sex- (*T*_b_ and *T*_pref_) and size- (performance curves) dependent.

Body size influences the range of possible *T*_b_s attainable from the environment by a lizard (Stevenson 1985). Thus, males and females of species like *S. jarrovii* that exhibit SSD may also differ in *T*_b_. In our study system, only male *S. jarrovii* are territorial during the summer (Moore and Marler 1987; MSL, unpubl. data). Territorial defense may provide males with access to higher-quality microhabitats than females with respect to the available thermal environment (e.g., Santos et al. 2008). Although we predicted that male field-active *T*_b_s should overlap with their *T*_pref_, we found little support for this prediction. Both males and females were captured at lower *T*_b_s than their *T*_pref_ in the laboratory. It may be that territorial defense provides minimal thermal benefits outside of the fall breeding season, when both sexes defend territories (Ruby 1978). The *T*_b_s we recorded are similar to those for *S. jarrovii* in Mexico (mean ± SE for adult lizards: 31.6 ± 0.2°C, Gadsen and Estrada-Rodriguez 2007). However, in our population, female *S. jarrovii* have higher *T*_b_s than males during the summer. Intersexual differences in *T*_b_ may be common in species exhibiting SSD (Lailvaux 2007), and a similar female-biased pattern has been detected in at least one other species of *Sceloporus*, *S. gadoviae* (Woolrich-Piña et al. 2012).

In addition, female *S. jarrovii*, unlike males, selected different temperatures when fed versus fasted in our study. Male *S. jarrovii* exhibited similar values for *T*_pref_ regardless of their digestive state. Body size had no effect on the *T*_pref_ of either sex. Previous studies have suggested that fasted lizards should select lower temperatures than fed lizards to conserve energy (Huey 1982). Selection of lower temperatures when fasted has been supported in numerous taxa, including *Alligator mississippiensis* (Lang 1979), *Anolis carolinensis* (Brown and Griffin 2005), *Anaxyrus boreas* (Carey 1978), and *Glyptemys insculpta* (Dubois et al. 2008). Thermoregulatory behavior following feeding, however, is not universal across taxa (e.g., Sievert 1989; Knight et al. 1990), and our experiment suggests that this phenomenon may vary within a species (i.e., between the sexes). Another study based on *S. jarrovii* from the Chiricahua Mountains in Arizona showed that male lizards selected similar temperatures when fed compared to when fasted for 48 h (Schuler et al. 2011). By not selecting lower temperatures when fasted, male *S. jarrovii* may prefer temperatures that favor multiple physiological and behavioral functions (Angilletta et al. 2002a), such as stamina, sprint speed, or display behavior (Schuler et al. 2011).

Lizards often use flight as a behavior for evading predators. Several studies have demonstrated that locomotor capacity and *T*_b_ are correlated in lizards (Bennett 1980). Locomotor performance may be maximized across a range of *T*_b_s in lizards (Angilletta et al. 2002a), and this pattern was supported for both male and female *S. jarrovii* in our study. As in previous studies, stamina was both temperature- and size-dependent. Larger lizards exhibited greater stamina, regardless of sex. Interestingly, the *T*_opt_ for stamina was significantly lower than the *T*_pref_ for both males and females. *Sceloporus jarrovii* is a viviparous species that breeds in the fall when ambient and *T*_b_s rarely reach the lower limits of those preferred in the laboratory in the current study (Tinkle and Hadley 1973; Beuchat 1989). Enhanced performance at lower *T*_b_s may therefore reflect an adaptation for cooler montane environments and in particular favor the maintenance of reproductive activity, territorial defense, and antipredator behavior during the breeding season.

## Conclusions

Multiple studies have demonstrated that morphological variation within a population is frequently associated with divergence in other physiological and ecological traits (Huyghe et al. 2007; Dreiss et al. 2012; Brazill-Boast et al. 2013; Hendry et al. 2014), including *T*_b_ (Jong et al. 1996; Hetem et al. 2009). Species that exhibit SSD are no exception (Brown and Weatherhead 2000; Butler et al. 2000). Our study suggests that for *S. jarrovii*, males and females also differ in aspects of their thermal physiology, including the degree to which those traits correlate with their differences in body size. More research into the extent of divergence in phenotypic traits between the sexes will add to our understanding of the ecological and evolutionary consequences of SSD.

Our findings are also consistent with previous analyses regarding the persistence of viviparous species in the face of climate warming (Sinervo et al. 2010). In particular, increasing ambient temperatures in the arid southwest of North America are predicted to displace higher-elevation forests with lower-elevation desert scrub and grassland vegetation types and their associated hotter and dryer environmental conditions (e.g., through altered precipitation regimes, see Brown et al. 1997). *S. jarrovii* is limited to elevations above 1300 m and breeds during the cooler fall months throughout this range (Tinkle and Hadley 1973). Our results reveal a physiological mechanism for the ability of *S. jarrovii* to remain active in cooler conditions and seasons. The *T*_opt_ for stamina is below *T*_pref_, but within the range of temperatures, lizards would experience during fall and winter. The predicted increase of 2–3°C (IPCC 2013) presents ectothermic species with two significant challenges. Based on our thermal performance curve, an increase in ambient temperatures of 2–3°C above *T*_opt_ for stamina results in 5–10% diminishment in performance. Because stamina has been shown to affect territory acquisition, social displays, and survivorship (Miles 1994; Sinervo et al. 2000; Miles et al. 2001), rising temperatures will have fitness and ultimately population growth consequences (Huey 1991; Clusella-Trullas et al. 2011). Second, the shift in habitat characteristics may affect the suitability of current locations (Chown et al. 2010). We also found that male and female *S. jarrovii* differ in field-active *T*_b_s. Body size, thermoregulation behavior, and heat balance interact such that larger animals tend to have greater thermal inertia and therefore exhibit slower heating and cooling rates than smaller animals (Stevenson 1985; Porter and Kearney 2009). The physiological and fitness consequences of climate warming may therefore differ for males and females of species with SSD. Male *S. jarrovii* may shoulder the heaviest burden from rising temperatures compared to females because of their larger body size and lower field-active *T*_b_s, at least during the summer. More data are needed to address this prediction, including whether fitness differences between the sexes are associated with variation in the relationship between *T*_b_ and body size within each sex. Regardless, the combination of multiple environmental, ecological, and physiological effects will likely enhance extinction rates for populations of *S. jarrovii* and other montane ectotherms in response to global climate change.

## Supporting Information

**Figure S1.** Adult female Yarrow's spiny lizard (*Sceloporus jarrovii*) peeking out over a boulder in the Huachuca Mountains in southeast Arizona, USA.

**Table S1**. Body size (snout-vent length, SVL) and body temperature data for *Sceloporus jarrovii* from this study and published datasets.
