# Supplementary figures and images for: Differences in the thermal physiology of adult Yarrow's spiny lizards (Sceloporus jarrovii) in relation to sex and body size
Source: Ecol Evol. 2014 Oct 20;4(22):4220–9. doi: 10.1002/ece3.1297 (PMC4267861; doi:10.1002/ece3.1297)

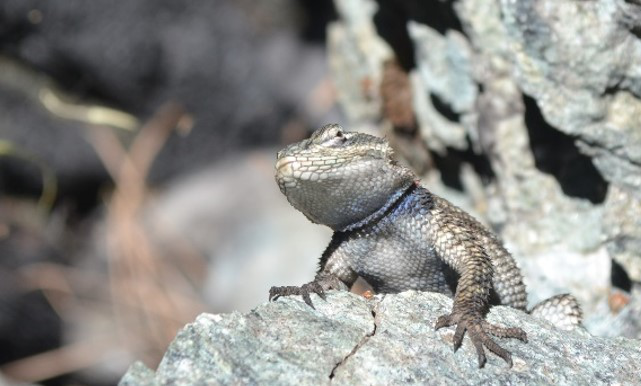

Supplement: Supplementary file 1 — Figure S1. Adult female Yarrow's spiny lizard (Sceloporus jarrovii) peeking out over a boulder in the Huachuca Mountains in southeast Arizona, USA. [file ece30004-4220-SD1.tif]
