# Supplementary material for: Differences in the thermal physiology of adult Yarrow's spiny lizards (Sceloporus jarrovii) in relation to sex and body size
Source: Ecol Evol. 2014 Oct 20;4(22):4220–9. doi: 10.1002/ece3.1297 (PMC4267861; doi:10.1002/ece3.1297)
Supplement: Supplementary file 2 — Table S1. Body size (snout-vent length, SVL) and body temperature data for Sceloporus jarrovii from this study and published datasets. [file ece30004-4220-SD2.docx]

**Table S1**. Body size (snout-vent length, SVL) and body temperature data for *Sceloporus jarrovii* from this study and published datasets. We only report mean values because the studies included in this table differ in statistics used to summarize trait variation (e.g., standard error, range). Percent sexual size dimorphism (SSD) is calculated as (SVL*_male_* – SVL*_female_*)/SVL*_female_* × 100% using mean values for each term and refers to how much larger on-average male lizards are than females (modified from Lovich & Gibbons 1992). Adult *S. jarrovii* at our study site exhibit male-biased SSD near the reported upper limit for the species (10% average but 18% max, Cox 2006).

| **Locality** | **N** | **Sex** | **SVL (mm)** | **Percent SSD (%)** | **Source** |
| --- | --- | --- | --- | --- | --- |
| Huachuca Mountains, AZ, USA | 25 | Male | 82.3 | 16 | *This study* |
|  | 34 | Female | 70.9 |  |  |
| Chiricahua Mountains AZ, USA | NR | Both | 70.6 | NR | *(Watters 2010)* |
| Chiricahua Mountains AZ, USA | 90 | Male | 93.4^a^ | 10 | *(Cox 2006)* |
|  | 86 | Female | 81.8^a^ |  |  |
| Arizona | 52 | Female | 71.8 | NA | *(Tinkle & Hadley 1973)* |
| Chiricahua Mountains AZ, USA | 501 | Male | 68.9 | 4.7 | *(Smith & Ballinger 1994)* |
|  | 645 | Female | 65.8 |  |  |
| Las Piedras Encimadas Canyon northwest of Durango, Mexico | 69 | Male | 69.8 | 5.8 | *(Gadsen & Estrada-Rodriguez 2007)* |
|  | 80 | Female | 66 |  |  |
| Mexico (preserved specimens at the National Autonomous University of Mexico [UNAM]) | 98 | Male | 73.5 | 4 | *(Ramírez-Bautista, Ramos-Flores & Sites Jr 2002)* |
|  | 62 | Female | 70.7 |  |  |

NR: Not reported. NA: Not available. ^a^ Values are means of asymptotic size (*A*) from growth models presented in Cox (2006) for each sex from the population at his high-altitude study site.

**References**

Cox, R. (2006) A test of the reproductive cost hypothesis for sexual size dimorphism in Yarrow's spiny lizard *Sceloporus jarrovii*. *Journal of Animal Ecology,* **75,** 1361-1369.

Gadsen, H. & Estrada-Rodriguez, J.L. (2007) Ecology of the spiny lizard *Sceloporus jarrovii* in the central Chihuahuan Desert. *The Southwestern Naturalist,* **52,** 600-608.

Lovich, J. & Gibbons, J.W. (1992) A review of techniques for quantifying sexual size dimorphism. *Growth, development, and aging: GDA,* **56,** 269-281.

Ramírez-Bautista, A., Ramos-Flores, O. & Sites Jr, J.W. (2002) Reproductive cycle of the spiny lizard *Sceloporus jarrovii* (Sauria: Phrynosomatidae) from north-central México. *Journal of Herpetology,* **36,** 225-233.

Smith, G.R. & Ballinger, R.E. (1994) Temperature relationships in the high-altitude viviparous lizard, *Sceloporus jarrovi*. *American Midland Naturalist***,** 181-189.

Tinkle, D.W. & Hadley, N.F. (1973) Reproductive effort and winter activity in the viviparous montane lizard *Sceloporus jarrovi*. *Copeia***,** 272-277.

Watters, J. (2010) A test of optimal foraging and the effects of predator experience in the lizards *Sceloporus jarrovii* and *Sceloporus virgatus*. *Behaviour,* **147,** 933-951.
